# Folding Assessment of Incorporation of Noncanonical Amino Acids Facilitates Expansion of Functional‐Group Diversity for Enzyme Engineering

**DOI:** 10.1002/chem.202002077

**Published:** 2020-09-04

**Authors:** Ivana Drienovská, Matúš Gajdoš, Alexia Kindler, Mahsa Takhtehchian, Barbara Darnhofer, Ruth Birner‐Gruenberger, Mark Dörr, Uwe T. Bornscheuer, Robert Kourist

**Affiliations:** ^1^ Institute of Molecular Biotechnology Graz University of Technology Petersgasse 14 8010 Graz Austria; ^2^ Diagnostic and Research Institute of Patholoy Diagnostic and Research Center of Molecular Medicine Medical University of Graz Neue Stiftingtalstraße 6 8010 Graz Austria; ^3^ Institute of Chemical Technologies and Analytics Vienna University of Technology Getreidemarkt 9/164 1060 Wien Austria; ^4^ Omics Center Graz BioTechMed-Graz Stiftingtalstraße 24 8010 Graz Austria; ^5^ Biotechnology & Enzyme Catalysis Institute of Biochemistry Greifswald University Felix-Hausdorff-Str. 4 17487 Greifswald Germany

**Keywords:** biocatalysis, enzyme expression, noncanonical amino acids, protein engineering, *pseudomonas fluorescens* esterase

## Abstract

Protein design is limited by the diversity of functional groups provided by the canonical protein „building blocks“. Incorporating noncanonical amino acids (ncAAs) into enzymes enables a dramatic expansion of their catalytic features. For this, quick identification of fully translated and correctly folded variants is decisive. Herein, we report the engineering of the enantioselectivity of an esterase utilizing several ncAAs. Key for the identification of active and soluble protein variants was the use of the split‐GFP method, which is crucial as it allows simple determination of the expression levels of enzyme variants with ncAA incorporations by fluorescence. Several identified variants led to improved enantioselectivity or even inverted enantiopreference in the kinetic resolution of ethyl 3‐phenylbutyrate.

## Graphical abstract

**In enzyme engineering** using an expanded genetical code, quick identification of fully translated and correctly folded variants is crucial. Herein, we report the engineering of the aryl esterase from *Pseudomonas fluorescens* utilizing a pool of ncAAs. Key for the identification of active and soluble protein variants was the use of the split‐GFP method, allowing for quick identification of interesting variants.

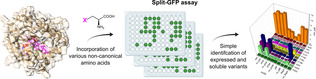

While a diversification of the limited set of functional groups in enzymes would greatly expand the possibilities of enzyme engineering, available methods for the incorporation of chemical entities to proteins are very limited.^1^ Post‐translational modifications of proteins typically rely on enzymatic steps with narrow substrate spectra and often require purified enzymes, that is usually too expensive for industrial biocatalytic applications.^2^ In contrast, incorporation of functional groups via the ribosomal protein machinery is, in principle, scalable. In general, ncAAs incorporation can be categorized in the modification of the protein backbone and the introduction of new functionalities in side‐chains.^3^ The in vivo incorporation of ncAAs either by sense‐codon reassignment (SCR) or stop‐codon suppression (SCS) has already been applied for the stabilization of enzymes,^4^ the modification of the enzymatic catalytic properties,^5^ and even the generation of new catalytic activities.^6^ In terms of biocatalysis, ncAAs can also be utilized for site‐specific immobilization.^7^ Additionally, this technology can be used to enhance/improve enzyme performance in hostile environments.^8^ Despite of these very promising examples, the biocatalytic use of enzymes containing non‐canonical amino acids (further referred to as allozymes) lacks behind the use of ncAAs as tools for therapy and research. In particular, ncAA incorporation is far from being a standard method for enzyme engineering.1c, 9 Furthermore, it should be noted that ncAA incorporation poses several different challenges. For de novo enzyme generation, usually spacious scaffolds with large pockets or cavities are chosen, and ncAA incorporation focuses on one site where the practicability of SCS has been proven. The use of ncAA for classical rational protein design, however, needs to incorporate novel functional groups into a tightly packed protein scaffold and to embed the ncAAs in the complex network of interactions between the amino acid residues. As the success of SCS is highly context‐dependent,^10^ application of ncAAs for enzyme engineering creates the demand for rapid methods to successfully determine ncAA incorporation and soluble production of the resulting allozymes (Figure 1).

**Figure 1:**
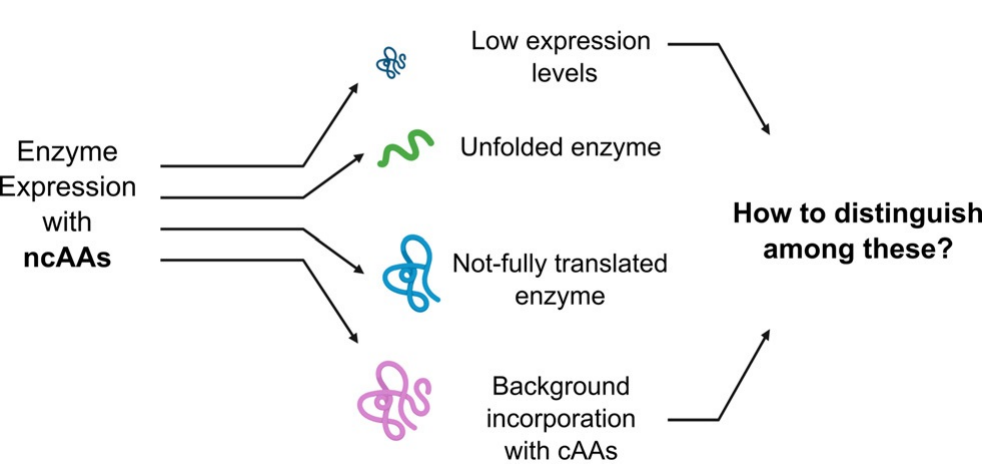
Technical hurdles of enzyme expression with ncAAs, which need to be addressed for the identification of soluble, active and functional enzymes.

The reason lies in the insufficient efficiency of the incorporation on the one hand (resulting in protein yields that are too low for application), and the limited flexibility on the other hand. Incorporation via SCS bears the risk of an abortion of the translation, leading to a high concentration of inactive enzyme fragments. The incomplete translation is controlled by active processes such as the recruitment of the release factor by the amber codon^11^ and depends on the position in the peptide chain (incorporations closer to the N‐terminal part being most successful), the ncAA and the surroundings of its position in the three‐dimensional structure. SCR leads to global substitution of a non‐canonical amino acid into all positions of a chosen canonical amino acid. Although examples of successful engineering utilizing this method has been showed, the need to substitute all residues poses some limitations for the engineering of proteins with a large number of the target residue.^12^ Finally, the SCR and SCS were successfully combined to produce one enzyme, however this approach also did not lead to eliminations of given drawbacks.^13^

Protein purification using a C‐terminal affinity tag is often used to remove fragmented proteins and to detect incorporation problems. Yet, affinity purification is rather unpractical for high‐throughput screens and severely reduces the throughput of the assay. The split‐GFP technique was developed by Waldo et al.^14^ and constitutes an easy way to verify production and successful folding of a protein. The technique was successfully adapted by some of us for rapid detection of soluble enzyme production levels for directed enzyme evolution.^15^ As incomplete translation is one of the main obstacles for the engineering of allozymes, we envisioned that this method might be highly practical to determine the amount of fully translated enzyme variants in high‐throughput screens with ncAAs. Prescreens and selection assays relying solely on activity of the enzyme would sort out misfolded variants, but usually overlook correctly folded variants with low specific activity. These variants are often of high interest for properties such as selectivity and can be valuable to increase knowledge and to provide starting points for further engineering. This is further complicated by the generally lower expression levels of proteins with ncAAs.

We expected that a split‐GFP assay coupled to an activity screen would allow discriminating between misfolded variants that showed neither fluorescence nor enzymatic activity, and correctly folded variants that gave a signal with split‐GFP, but showed little activity. Control cultivations without addition of the ncAA can easily highlight promiscuous incorporation of canonical amino acids. To demonstrate the feasibility of the assay, we investigated the rational engineering of the enantioselectivity of a *Pseudomonas fluorescence* esterase (PFE)^16^ in the kinetic resolution of *rac*‐ethyl 3‐phenylbutyrate by incorporation of a set of five different ncAAs. Using the split‐GFP assay in combination with an activity test allows the quick identification of folded and active variants with interesting properties and decreases the need for time‐consuming screenings of all prepared variants (Figure 2). Ten residues in the active site region of PFE were selected as positions for ncAA incorporation. Enantioselectivity strongly depends on the difference of transition state energy in the conversion of both enantiomers of the substrate, which is influenced by steric hindrances, hydrogen bond formation, charge and π–π interactions. We believe that ncAAs can provide these effects beyond their canonical counterparts. Therefore, the residues surrounding the catalytic triad, or forming the acyl or alcohol binding pocket were selected (Figure 3).

**Figure 2:**
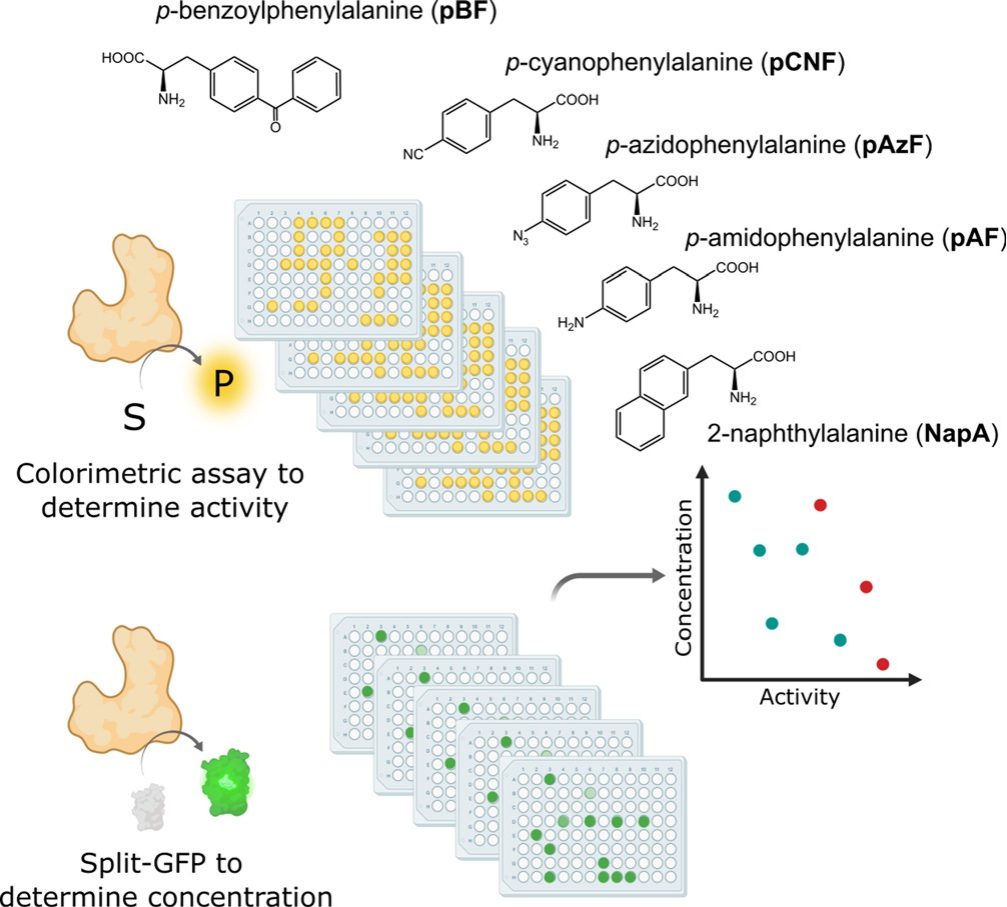
Schematic representation of the envisioned work‐flow of the proposed methodology comprising the detection of esterase activity by the colorimetric pNPA assay and determination of PFE protein content using the split‐GFP method. From these data the specific activity can be calculated and normalized. Furthermore, the chosen ncAA are shown.

**Figure 3:**
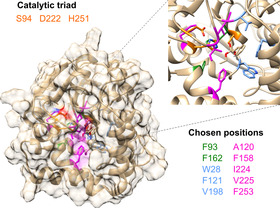
Surface view of the PFE protein (PDB: 1VA4) and a close‐up of the active site pocket. In both representations, the positions that were used for the introduction of ncAAs are highlighted in color (green for the alcohol‐binding site, blue for the acyl‐binding site and magenta for surrounding residues) and the catalytic triad is highlighted in orange. The same color coding was used in the list of chosen positions shown in the Figure.

The selection of residues for incorporation was led mainly by the structure of PFE, with previous reported studies on the engineering of this enzyme taken into consideration.^17^ The side chains of Trp28 and Val225 lie on either side of the acyl binding region, while the side chains of Val121 and Phe198 lie above and behind the acyl binding region. Residues F93 and F162 form an alcohol‐binding pocket. The substrate‐binding site of PFE is almost exclusively hydrophobic with numerous aromatic residues, therefore the hydrophobic character was also kept in incorporation of ncAA. However, we decided to incorporate additional features, in terms of side chain bulkiness as well as additional interactions to pursue novel binding modes and subsequent favoritism of one substrate stereoisomer via size restrictions and aromatic systems π–π stacking between the ncAA and the substrate. The chosen ncAAs are summarized in Figure 2. The amber stop codon suppression methodology was used to introduce this set of ncAAs into all the selected position (Supplementary Table 1).^18^ To successfully apply the split‐GFP method for the screening of protein expression, the protein of interest must be expressed with the GFP11 fragment as a fusion tag. The cloning of the PFE gene with the split‐GFP tag as well as incorporation of the amber stop codon was performed as described in the method section of the Supplementary material. Ten variants and wild‐type PFE and the corresponding plasmids containing the orthogonal translation system for the introduction of the ncAAs were transformed into *E. coli* and coexpressed in deep‐well plates; all experiments were performed in triplicates. Negative controls were grown identically, without addition of the ncAAs. Hence, if present, the unspecific incorporation of canonical AAs could be observed and screened for. Selection of active variants of PFE was done based on the pNPA activity assay monitoring the hydrolysis of the acyclic ester *p*‐nitrophenyl acetate **1** (Scheme 1 a). The activity determined in the cell free extract (CFE) was related to the protein content determined by fluorescence measurement employing the GFP‐split assay (see Supporting Information for more details) and compared to the activity of the wild‐type PFE (wtPFE). Results of the split‐GFP assay and the activity tests are summarized in Figures 4 and 5 and in more details in the Supporting Information.

**Table 1:** Enantiomeric ratios obtained for selected PFE variants. The calculated enantiomeric ratios (E) are shown, calculated from all measurements with conversion in between 10–80 %, with enantiomeric excesses of products (*ee*_p_) and the respective conversion (*c*) given for 4 hours measurement.

PFE	Conv. [%]^[a]^	*ee_p_* [%]^[a]^	*E* ^[b]^
wt	72	27	(*R*) 2.3
F158_pAzF	5	35	(*R*) 2.6
F158_NapA	14	47	(*R*) 3.0
F162_NapA	23	68	(*R*) 5.8
F198_pAzF	26	58	(*R*) 4.4
F198_NapA	9	47	(*R*) 2.7
I224_pAzF	18	21	(*S*) 1.9

[a] Determined by GC‐FID analysis. [b] Calculated according to Chen et al.^20^

**Scheme 1:**
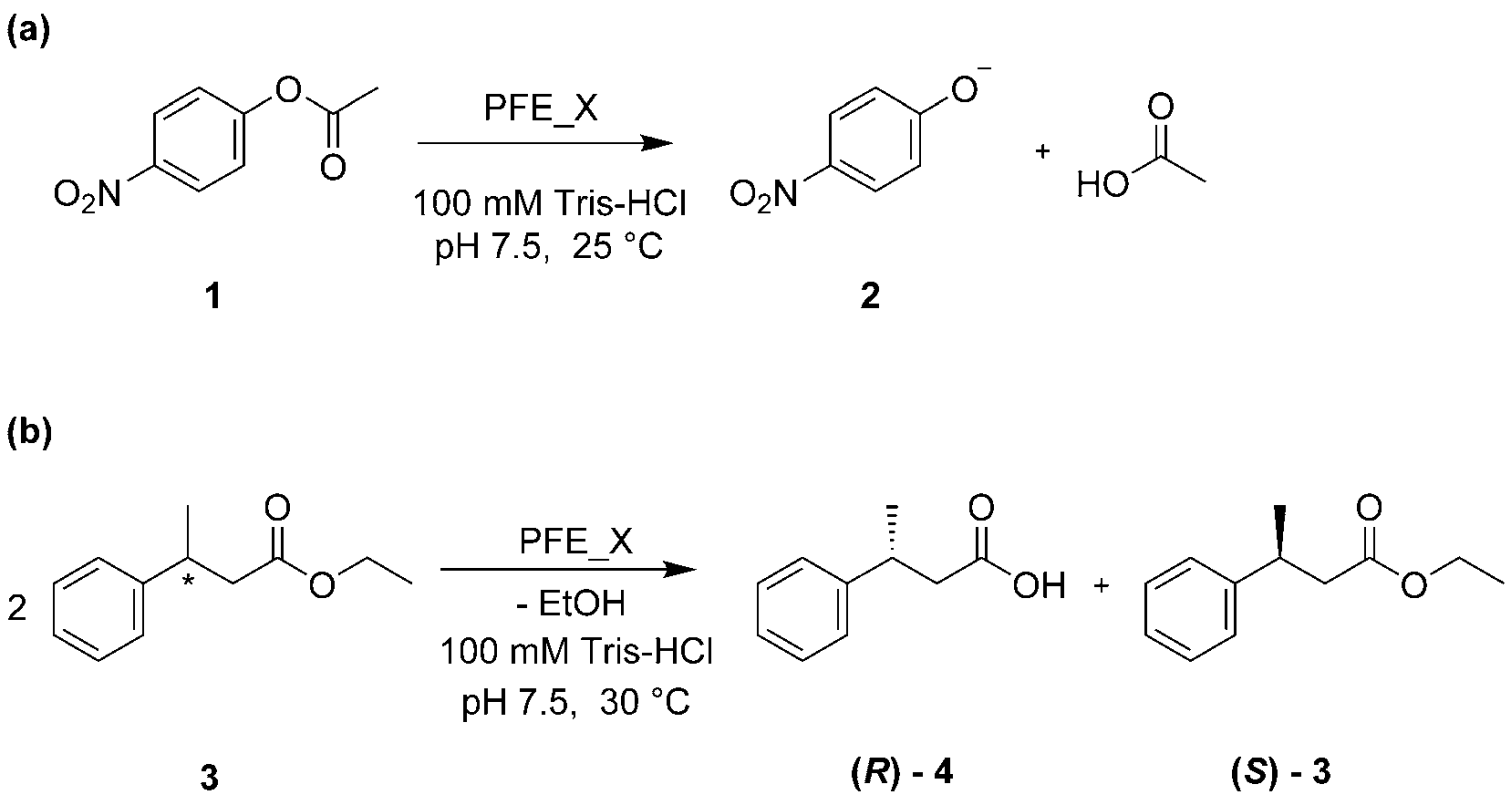
(a) pNPA assay, hydrolysis of *p*‐nitrophenylacetate **1** to *p*‐nitrophenolate **2** and acetic acid. The progress of the reaction was monitored following the increase in absorbance at 410 nm due to the formation of **2**. (b) Kinetic resolution of *rac*‐ethyl 3‐phenylbutyrate **3**. The progress of the reaction was monitored by gas chromatography analysis using a chiral column. X in PFE_X represents different mutant variants of PFE.

**Figure 4:**
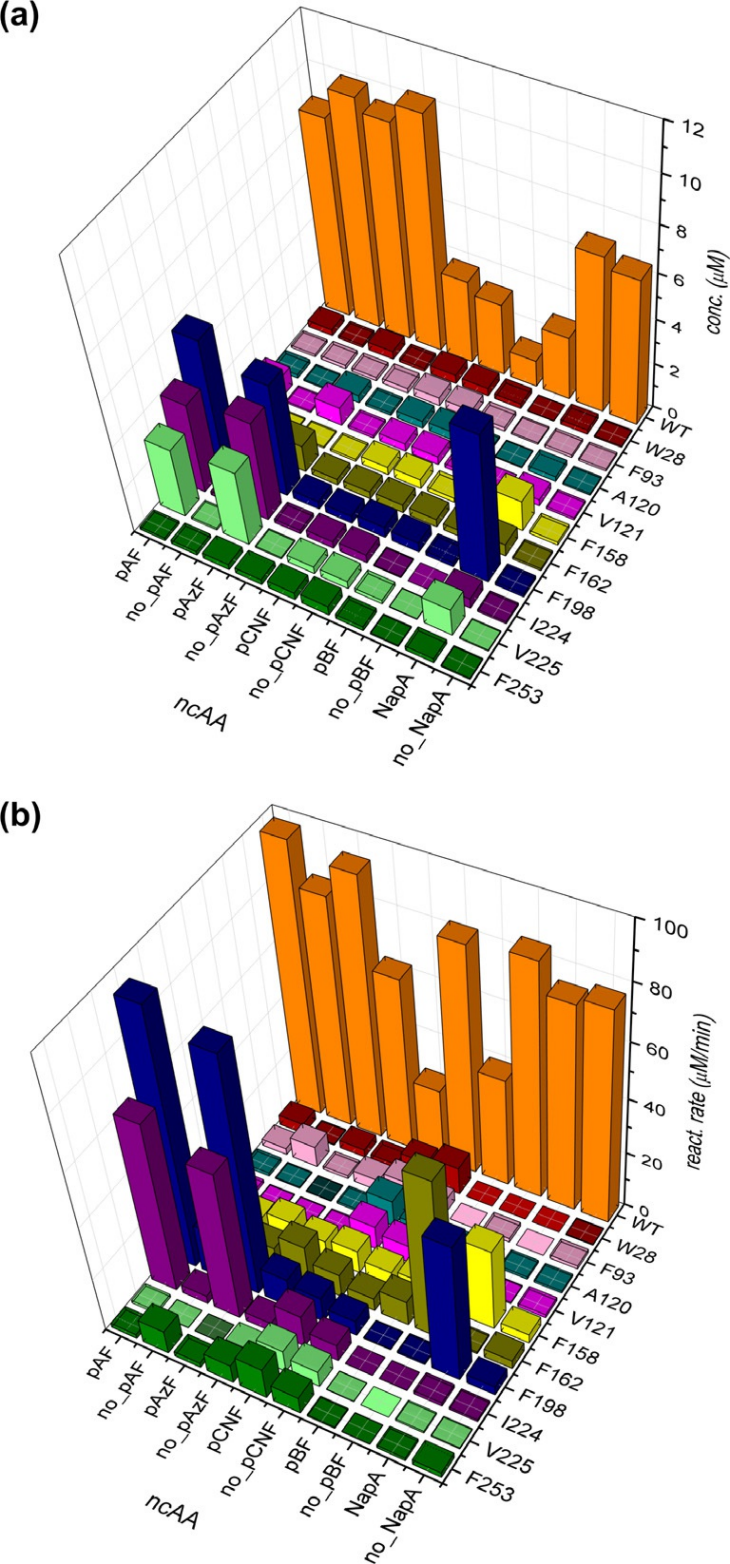
Graphic 3D representation of a) expression levels of PFE variants measured in cell‐free extracts (in μm) using the split‐GFP assay and b) corresponding reaction rates measured for pNPA assay (in μm min^−1^). Conditions with added ncAA (pAzF, pBF, pCNF, NapA) during expression are compared with control experiments, where ncAA was not added (no_pAzF, no_pBF, no_pCNF, no_NapA). For details on expression and reaction conditions see Supporting Information.

The overall results of these experiments (Figure 4 a) show that the incorporation of different ncAAs is not well tolerated in the PFE scaffold at multiple positions, underlining the need for an efficient method for the identification of well‐produced variants. Out of the ten positions selected, four (W28, F93, A120 and F253) gave no or minimal levels of soluble protein, independent of the studied ncAA. Positions V121, F158 and F162 resulted in low expression, depending on the studied ncAA, and positions F198, I224 and V225 were generally well tolerant for ncAA incorporation. When looking at the specific ncAAs, three of them (pAF, pAzF and NapA, see Figure 2) were well incorporated, while limited amounts of expressed, soluble enzyme were observed for the remaining two (pBF, pCNF). The expression levels of most pBF‐carrying PFE variants were not significantly higher than the background incorporation and showed no detectable activity. pBF has a significantly bulkier side chain compared to pAzF or NapA, which may cause the inability of the enzyme to fold correctly, or the active site accessibility is too restricted. Secondly, the minimal levels of soluble protein were also observed in case of incorporation of pCNF. Herein, a different vector system pULTRA_pCNF was used for incorporation of pCNF. In the conditions used for the incorporation of pCNF, undesired background incorporation was significantly higher compared to the other systems described in this study (i.e. the pEVOL‐based plasmids). Interestingly, in the case of these two ncAAs (pBF, pCNF), lower levels of expression/amount of soluble protein of wtPFE were also observed, with almost 10x reduction in expression for pBF suggesting that these systems may have significant effects on the general „healthiness“ of the expression machinery. PFE variants with pAzF at position F162, F198, I224 and V225 were well expressed, with F198_pAzF and I224_pAzF having specific enzyme activities higher than the wtPFE. The pAF incorporation was achieved by the reduction of pAzF after expression, therefore similar trends are observed for incorporation levels, while allowing us to study effects of different ncAAs on reaction rates and specific activity. Out of the ten PFE variants, the NapA substitution at positions F158, F198 and V225 gave significant expression levels, with high specific esterase activity in case of F198_NapA, albeit slightly lower than the wt (Figures 4 and 5).

**Figure 5:**
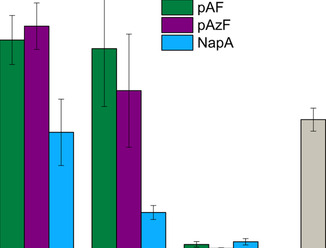
Comparison of specific enzyme activities calculated using the pNPA assay for PFE variants with three different non‐canonical amino acids at three different positions compared to the wtPFE. Error‐bars stem from three independent cultivations.

All variants selected for further studies were produced in *E. coli* and purified by metal affinity chromatography (see the Supporting Information, Figure S1 and S2). Typical purification yields were in the range of 28–72 mg L^−1^, generally lower than the expression yield of wild‐type PFE (105–120 mg L^−1^). The expression levels of purified proteins were consistent with the results from the small‐scale experiments using the split‐GFP methodology, in‐between 25–64 % in comparison to the wild type (100 %). The level of expression was strongly dependent on the ncAA itself, as well as on the position in the sequence. The incorporation of ncAAs in the proteins were confirmed with electrospray ionization mass spectrometry (ESI‐MS), at different studied positions in at least one variant per unnatural amino acid. No peaks corresponding to alternative amino acid incorporation were observed (Figure S3). The split‐GFP method is therefore straightforward in identifying any variants where promiscuous incorporation is a potential problem. These results, together with our negative controls for small‐scale screening suggests no (or minimal) background incorporation of natural amino acids.

The effect of the ncAAs on the activity and enantioselectivity were further studied using the kinetic resolution of ethyl 3‐phenylbutyrate (**3,** Scheme 1 b). Results are summarized in Table 1. In several cases, significant effects on enantioselectivity were observed. A significant increase in optical purity (% *ee*) was achieved by substituting F198 by pAzF and F162 by NapA. The substitution of F198 with pAzF nearly doubled the enantiomeric ratio in the kinetic resolution of **3** showing preference for the (*R*)‐enantiomer. The enantioselectivity increase was even stronger when F162 was substituted by NapA. Residue F198 had already been reported in literature to be a hot‐spot for enantioselectivity of PFE.17b, 17d This residue forms the acyl‐binding pocket of the enzyme and is in direct proximity with the substrate, around 3.5 Å from the aromatic ring of the **3**. On the other hand, a mutation of the F162 residue, based on our knowledge, has not been reported yet. This residue is part of the alcohol‐binding pocket. The mechanism, whose consequence is improvement of enantioselectivity, is probably indirect through a rearrangement of neighboring amino acid side chains. A substitution at position I224 has already yielded a switch in enantioselectivity in a previous report.17d In the reported study, the substitution I224F led to an E of 1 for (*S*)‐**4** in the kinetic resolution of **3**. In our study, the variant I224pAzF_PFE enabled almost a doubling in the enantiomeric ratio. A direct influence of I224 on the enantioselectivity was also strongly expected since its side chain is in direct contact with the acyl moiety of the substrate on the opposite side to F198. Substitution for aromatic side chains possibly enables π interaction that leads to the different binding mode and subsequent preference of the (*S*)‐enantiomer. This supports the previous claims that bulky and hydrophobic amino acids, which block the entrance to the active site of the PFE have effect on selectivity. Overall, our data showcases that utilizing ncAAs provides another means to redesign the active site next to standard directed evolution techniques, where a switch of enantioselectivity can also be achieved.^19^

In summary, we report the engineering of the aryl esterase from *Pseudomonas fluorescens* utilizing a pool of ncAAs. Altogether five different aromatic and bulky ncAAs were used to remodel the binding pocket of PFE, at ten different sites. The resulting 50 PFE variants were expressed and their activity and foldability were assessed. The PFE variants, which were selected as attractive based on their activity and foldability, underwent enantioselectivity screening using the ethyl ester of 3‐phenylbutyrate in a kinetic resolution. Substitution of F198 for pAzF and F162 for NapA led to improved enantioselectivity of the enzyme towards (*R*)‐**3**. Substitution of I224 for pAzF swapped the enantiopreference of the enzyme for (*S*)‐**3** and outperformed the canonical amino acid substitution of I224Y, in enantiomeric ratio by a factor of two. Our data demonstrate that the split‐GFP self‐assembly method facilitates high‐throughput screening of production levels of an ncAA‐incorporated enzyme. The speed and simplicity of the method allow for easy screening of expression levels, reliable elimination of misfolded variants and those with incomplete translation, as well as estimation of ncAA‐carrying enzyme concentration in cell‐free extracts. We expect that this method will widely extend the capacity to test ncAA‐carrying enzyme variants in a short time and thus to overcome obstacles for wide application of ncAA for enzyme engineering. Ultimately, we showcase that the power of the method expands beyond traditional directed evolution screenings to libraries of ncAA‐carrying enzymes.

## Conflict of interest

The authors declare no conflict of interest.

## Supporting information

As a service to our authors and readers, this journal provides supporting information supplied by the authors. Such materials are peer reviewed and may be re‐organized for online delivery, but are not copy‐edited or typeset. Technical support issues arising from supporting information (other than missing files) should be addressed to the authors.

Supplementary
