# Supplementary material for: Folding Assessment of Incorporation of Noncanonical Amino Acids Facilitates Expansion of Functional‐Group Diversity for Enzyme Engineering
Source: Chemistry. 2020 Sep 4;26(54):12338–42. doi: 10.1002/chem.202002077 (PMC7590180; doi:10.1002/chem.202002077)
Supplement: Supplementary file 1 — Supplementary [file CHEM-26-12338-s001.pdf]

Chemistry–A European Journal

Supporting Information

Folding Assessment of Incorporation of Noncanonical Amino Acids Facilitates Expansion of Functional-Group Diversity for Enzyme Engineering

Ivana Drienovská,[a] Matúš Gajdoš,[a] Alexia Kindler,[a] Mahsa Takhtehchian,[a] Barbara Darnhofer,[b, d] Ruth Birner-Gruenberger,[b, c, d] Mark Dörr,[e] Uwe T. Bornscheuer,[e] and Robert Kourist*[a]

Supplementary Information

Folding assessment of incorporation of non-canonical amino acids facilitates expansion of functional group diversity for enzyme engineering

Ivana Drienovská[a], Matúš Gajdoš[a], Alexia Kindler[a], Mahsa Takhtehchian[a], Barbara Darnhofer[b][d], Ruth Birner-Gruenberger[b] [c] [d], Mark Dörr[e], Uwe T. Bornscheuer[e] and Robert Kourist*[a]

[a]Institute of Molecular Biotechnology, Graz University of Technology, Petersgasse 14, 8010 Graz, Austria
[b]Diagnostic and Research Institute of Patholoy, Diagnostic and Research Center of Molecular Medicine, Medical University of Graz, Neue Stiftingtalstraße 6, 8010 Graz, Austria
[c]Institute of Chemical Technologies and Analytics Vienna University of Technology, Getreidemarkt 9/164, 1060 Wien, Austria
[d]Omics Center Graz, Biotechmed-Graz, Stiftingtalstraße 24, 8010 Graz, Austria
[e]Biotechnology & Enzyme Catalysis, Institute of Biochemistry, Greifswald Universitity, Felix-Hausdorff-Str. 4, 17487 Greifswald, Germany

*Corresponding author: kourist@tugraz.at

Table of contents

1. Material and Methods S3

2. Supplementary Tables S9

3. Supplementary Figures S11

4. References S18

1. Material and Methods

General remarks

Unless stated otherwise, all chemicals, solvents and buffers were purchased from *SigmaAldrich* (Germany) or *Carl-Roth GmbH* (Germany) and used without further purification. Restriction enzymes and polymerases were purchased from *Thermo Fisher* (Germany) and *New England Biolabs* (Germany). Non-canonical amino acids were purchased as racemic mixtures from *Acrotein ChemBio* (USA). *E. coli* strains XL-1-Blue, BL21(DE3) and TOP10 were used for cloning and expression. DNA sequencing was carried out by *Microsynth AG* (Switzerland). Primers were synthesized by *Eurofins MWG Operon* (Germany).

Cloning of pBAD_PFE_GFP11

50 µL polymerase chain reaction (PCR) mixture consisted of: 1 µL of template plasmid DNA solution (pJOE2792_PFE, ~ 50 ng/µL), 0.5 µL of forward primer and 0.5 µL of reverse primer (both 100 µM) (**Supplementary Table 2_1-2)**, 1 µL of dNTPs (2 mM each), 10 µL of Q5 Buffer (5X), 32.5 µL double-distilled water, and 1 µL of Q5 DNA polymerase (*New England Biolabs,* Germany). The following PCR procedure was used: Initial denaturation at 95 °C for 30 seconds, followed by 25 cycles with denaturation at 98 °C for 20 seconds, annealing at 63 °C for 20 seconds and extension at 72 °C for 1 minute. The final step of extension at 72 °C for 10 minutes. The product was then isolated by cutting the band of interest from the 1 % agarose gel after the electrophoresis, (the gel extraction kit, *Thermo Fisher*, Germany). After obtaining the insert, both insert and vector pBAD_GFP11_T7LysH17A were digested with restriction enzymes *Xho I* and *Spe I* (*Thermo Fisher*, Germany). 40 µL mixture contained: 25 µL of a vector/insert, 1 µL of each restriction enzyme, 4 µL of FastDigest Green Buffer and 9 µL of double-distilled water. The reaction took place at 37 °C overnight. Fragments of interest were then separated by electrophoresis on a 1 % agarose gel. Cut DNA fragment was isolated from the gel band using the gel extraction kit (*Thermo Fisher*, Germany). 20 µL ligation reaction mixture contained: 7 µL of cut insert DNA solution, 2 µL of cut vector DNA solution, 10 µL of T4 DNA reaction buffer (2X) 1 µL T4 DNA ligase (*Thermo Fisher*, Germany). Such reaction mixture was incubated overnight at 16 °C. After incubation, 5 µL from the mixture was used to transform *E. coli* TOP10 cells.

QuikChange PCR – Site-Directed Mutagenesis Method

50 µL QuikChange™ polymerase chain reaction mixture consisted of: 1 µL of template plasmid DNA solution (pBAD_wtPFE, ~ 5-15 ng/µL), 0.5 µL of forward primer and 0.5 µL of reverse primer (both 100 µmol/L), 1 µL of dNTPs (2 mmol/L) (*Thermo Fisher*, USA), 1.5 µL of dimethylsulfoxide (DMSO), double-distilled water, 5 µL of *Pfu* 10x buffer and 1 µL of *Pfu* DNA polymerase. The following PCR procedure was used: Initial denaturation at 95 °C for 30 seconds, followed by 25 cycles with denaturation at 95 °C for 20 seconds, annealing at 53 - 65 °C for 30 - 60 seconds. Annealing temperature was set based on the melting temperature of a primer; 3 – 5 °C lower than the melting temperature. Extension at 72 °C for 7 min. Final step of extension at 72 °C for 10 minutes. 5 µL from the mixture were used to directly transform *E. coli* XL-1-Blue cells and successful mutation was confirmed by sequencing. The list of primers used is given in **Supplementary Table 2 (3-21).**

Small-scale expression in 96-well format

In each well of the autoclaved 96-deep well plates (DWPs) 950 µL of LB medium (*Carl Roth,* Germany) with appropriate antibiotics (**Supplementary Table 1**) were added. 50 µL of overnight cultures were added as the inoculum. Cultures were grown at 400 rpm at 37 °C. After 4.5 h growth, when OD reached value between 0.6-1, inducers and respective non-canonical amino acid were added (**Supplementary Table 1**). Then, DWPs were placed to the shaker at 28 °C, 400 rpm for the overnight expression. After the expression, cultures in DWPs were pelleted in the centrifuge (Eppendorf, Germany) (3220 g, 30 min, 4 °C). Pellets were resuspended in 200 µL of BugBuster® Master Mix (*Merck Millipore,* Germany), then incubated at the 28 °C on the shaker for 2 hours and freeze – 20 °C until further used. For the pAF lysates, 20µl of Tris(2-carboxyethyl)phosphine stock solution (100 mM) was added and the reduction was performed for an additional 2 hours at 30 °C. The resuspended cells were frozen -20 °C till further used Cell-free extracts were obtained as supernatants after centrifugation (3220 g, 60 min, 4 °C) and transferred to fresh microtiter plates (MTPs).

p-nitrophenylacetate assay

In a MTP, 10 µL of cleared lysates were mixed with 180 µL of Tris-HCl buffer (100 mM Tris-HCl, 150 mM NaCl, pH 7.5). Absorbance over time (λ = 410 nm) was measured in the

spectrophotometer (*BioTek*, USA) immediately after the addition of 10 µL of 20 mM *p*-nitrophenyl acetate in DMSO. The PFE activity in a CFE was calculated based on the calibration as the initial reaction velocity ($\mu M^{-1}\ s^{-1}$).

Split-GFP Method

PFE variants were expressed with the GFP11 fragment as a fusion tag. Content (expression level) of such fusion proteins was determined using fluorescence (Santos-Aberturas *et al.*, 2018)[1]. In brief, 180 µL of reporter solution (see *Preparation of GFP 1-10 Reporter Solution*) were mixed in a MTP with 20 µL of CFE. The MTP was incubated for 18 hours at 4 °C then fluorescence (λex = 488 nm/ λem = 530 nm) was measured in the spectrophotometer (*BioTek*, USA).

Large Scale Protein Expression and Protein Purification

In an autoclaved Erlenmeyer flask with 0.5 L of LB medium (10 g/l peptone, 5 g/l yeast extract, 10 g/l NaCl) with respective antibiotics (**Supplementary Table 1**) 5 mL (1 % (v/v)) inoculum from an overnight culture was added. The culture was grown under agitation at 37 °C. After ~4 h growth, when OD reached value between 0.4 – 0.6, inducers (**Supplementary Table 1**) and the respective non-canonical amino acid (1 mM final concentration) were added. Then, the flask was placed to the shaker at 28 °C for the overnight expression. After the expression, culture was transferred to a centrifuge beaker and pelleted in the centrifuge (*Beckman Coulter*, USA) (4000 g, 20 min, 4 °C). Pellets were resuspended in 20 mL of equilibration buffer (50 mM K_2HPO_4, 150 mM NaCl, 10% glycerol, pH 8.0). Cells suspension underwent sonication (6 min, 60% work/rest, 70% power input). Cell-free extracts (CFE) were obtained as the supernatants after the centrifugation (30 000 g, 60 min, 4 °C). A chromatography column with Ni-NTA resin (Ni Sepharose TM 6 Fast Flow, *GE Healthcare*, Sweden) was equilibrated with 10x column volume of equilibration buffer (50 mM K_2HPO_4,150 mM NaCl, 10% glycerol, pH 8.0) and loaded with the CFE. Ni(II) – His-tag affinity interactions were facilitated during 30 min incubation at 4 °C. The columns were washed with 6x CV of wash buffer (50 mM K_2HPO_4, 150 mM NaCl, 30 mM imidazole, 10% glycerol, pH 8.0). Elution was carried out with elution buffer (50 mM K_2HPO_4, 150 mM NaCl, 300 mM imidazole, 10% glycerol, pH 8.0). After purification, the enzyme was rebuffered to Tris-HCl buffer (100 mM Tris-HCl, 150 mM NaCl, pH 7.5) using ultrafiltration (*Merck Millipore*, Ireland) or dialysis (*Sigma-Aldrich*, Austria). Following the purification, the

fractions were analyzed by SDS-PAGE electrophoresis on 12% polyacrylamide gels (ExpressPlus™ PAGE Gel, *Genscript*, USA) followed by staying with Coomassie Brilliant Blue. Protein concentration was determined by measuring absorbance at 280 nm on a *Thermo Scientific* Nanodrop 2000 UV–Vis spectrophotometer or using bicinchoninic acid assay (*Thermo Fisher*, Germany).

Enantioselectivity Screening

975 uL of CFE were mixed with 10 uL of 0.1 mol/L acetophenone (internal standard) and 25 uL of *rac*-ethyl 3-phenylbutyrate 0.8 mol/L (in DMSO) in a fresh 2 mL Eppendorf tube. The reaction proceeded in a thermomixer at 30 °C and 650 rpm. 200 µL samples were taken usually at the time point 4, 6, 8 and 24 h. Samples were extracted to 500 µL DCM (CH_2Cl_2). Then the reaction products ((*R*)- and (*S*)-3-phenyl butyric acid) were derivatized with 20 µL TMS-diazomethane and 50 µL MeOH. After 30 min incubation at laboratory temperature, the remaining part of TMS-diazomethane was removed by 10 µL of acetic acid. Afterwards, the mixture was dried in the vacuum drier (45 °C, 1 h). The dried fraction was dissolved in 200 µL DCM and used for the chiral GC measurement. The enantiomeric ratio (E) was calculated based on the enantiomeric excess of product (ee_p) and substrate (ee_s) from measurement where conversion reached between 10-80%.[2] Enantiomeric excess and conversion were calculated as functions of the peak areas, measured by chiral GC-FID. The results shown in Table 1 are average values obtained by at least two independent measurements (in case of PFE_F158NapA only a single measurement). Standard deviations are below 5%.

<u>GC-FID parameters used for the analytics</u>

Instrument: Nexis GC-2030 (*Shimadzu Europa* GmbH.)
Column: Hydrodex-β-TBDAc (*Macherey-Nagel*)
length: 50 m, inner diameter: 0.25 mm, film thickness: 0.25 µm
Injection volume: 1 µL
Injection temp.: 230°C
Injection mode: Split
Flow control mode: Velocity
Pressure: 121.3 kPa
Total flow: 101.5 mL / min
Column flow: 0.98 mL / min
Linear velocity: 22.9 cm / s
Purge flow: 3 mL / min
Split ratio: 100
Oven temp. program: 8 min at 70°C, 3°C per min to 120°C, 3 min at 120°C, 1°C per min to 150°C, 1 min at 150°C, 20°C per min to 220°C and 1 min at 220 °C
FID temperature: 250°C

Preparation of GFP 1-10 Reporter Solution

The reporter solution for fluorescence determination of expression level was prepared following the protocol by Santos-Aberturas *et al*.[1] with minor changes. 500 mL LB medium was inoculated by adding 5 mL of an overnight culture of *E. coli* BL21(DE3) containing plasmid pET41b_GFP (1-10) and placed to the shaker at 37 °C. After OD ≈ 0.6 had been reached, the protein expression was induced by adding IPTG to final concertation 0.1 mM. Then the culture was placed to the shaker at 28 °C for overnight expression of GFP1-10 inclusion bodies. After expression, the culture was centrifuged (4000 g, 20 min, 4 °C). Pelleted cells were resuspended in 20 mL cold TNG buffer (100 mM **T**ris/HCl, 150 mM **N**aCl, 10% (v/v) **G**lycerol) and transferred to fresh Falcon-type tube. Then the cells were disrupted by sonication (10 min, 50% work/rest, 50% power input). The produced suspension, which included GFP1-10 inclusion bodies was centrifuged at 30 000 g (30 min, 4 °C). The supernatant was discarded and the pellet that contained inclusion bodies was resuspended in 10 mL of BugBuster® Master Mix with the help of sonication (2 min, 50% work/rest, 50% power input). The generated suspension was centrifuged (30 000 g, 30 min, 4 °C), the supernatant discarded, and the pellet resuspended again in 10 mL of BugBuster® Master Mix (sonication: 2 min, 50% work/rest, 50% power input). This step (resuspension in BugBuster® Master Mix followed by centrifugation) was repeated two more times. To remove the detergent, the pellet was resuspended with the help of sonication in 10 mL of TNG buffer. Inclusion bodies were pelleted by centrifugation (30 000 g, 30 min, 4 °C). This step was repeated one more time. The obtained inclusion bodies were weighted and resuspended in TNG buffer to reach a concentration of 75 mg/mL. Sonication was implied again to stimulate the resuspension. This suspension was split to fresh tubes in 1 mL aliquots, centrifuged (16 000 g, 10 min, 4 °C), the supernatant was discarded, and pelleted inclusion bodies stored at -20 °C. Each aliquot was resuspended in 1 mL of urea solution (urea 9 mol/L and DTT 5 mmol/L). The insoluble fraction was removed by centrifugation (16 000 g, 1 min) and supernatant, which contained solubilized GFP1-10 fragments was added into 50 mL of buffer solution (100 mM Tris-HCl, 150 mM NaCl).

Exact Mass analysis

The protein solutions were desalted using 3 kD Amicon Ultra 0.5 mL Centrifugal filter units (*Millipore*, USA). A final protein concentration of 40 ng/µL in 2% acetonitrile and 0.1% formic acid (in water) was obtained. Protein species were separated by nano-HPLC (Dionex Ultimate 3000) equipped with a Pepswift precolumn (monolithic, 5 x 0.2 mm) and a ProSwift RP-4H column (monolithic, 100 µm x 25 cm) (all *Thermo Fisher Scientific*, Austria). 2 to 4 µl of protein sample was injected and concentrated on the enrichment column for 3 min at a flow rate of 5 µl/min with 0.1% formic acid as isocratic solvent. Separation was carried out on the nanocolumn at a flow rate of 1 µL/min at 37°C using the following gradient, where solvent A is 0.1% formic acid in water and solvent B acetonitrile containing 0.1 % formic acid: 0-2 min: 5% B; 2-17 min: 5-60% B; 17-20 min: 60% B; 20-20,1 min: 60-5% B; 20,1-29 min: 5% B. The maXis II ETD mass spectrometer (*Bruker*, Germany) was operated with the captive spray source in positive mode with following settings: mass range: 300 – 3000 m/z, 1 Hz, source voltage 1.3 kV, dry gas flow 3 L/min at 180°C. The protein mass spectra were deconvoluted by Bruker Data analysis software, using the MaxEnt2 algorithm. The following main parameters were applied: m/z range, min. 10,000 to max. 50,000, instrument resolving power was set to 50,000. For peak detection SNAP algorithm with following parameters were used: Quality factor threshold 0.9, S/N threshold 2.

2. Supplementary Tables

Supplementary Table 1. List of expression vector used in this study, with respective final inducer concentration in the media, their respective resistance selection markers and their final concentrations.

Expression vector	Gene/function	Induction (final concentration)	Selection Marker (final concentration)
**pJOE2792_PFE**	PFE enzyme	0.02 % Rhamnose	Ampicillin (100 μg/mL)
**pBAD_GFP11_T7LysH17A**	pBAD carrying GFP11 tag	0.02 % Arabinose	Ampicillin (100 μg/mL)
**pBAD_PFE_GFP11**	PFE – GFP_11 fusion TAG	0.02 % Arabinose	Ampicillin (100 μg/mL)
**pET41b_GFP(1-10)**	GFP (1 – 10) fragment	0.1 mM IPTG	Kanamycin (50 μg/mL)
**pEVOL_pAzF**	pAzF incorporation	0.02 % Arabinose	Chloramphenicol (34 μg/mL)
**pEVOL_pBF**	pBF incorporation	0.02 % Arabinose	Chloramphenicol (34 μg/mL)
**pEVOL_NapA**	NapA incorporation	0.02 % Arabinose	Chloramphenicol (34 μg/mL)
**pULTRA_CNF**	pCNF incorporation	1 mM IPTG	Spectinomycin (50 μg/mL)

For plasmids used within this study pEVOL_pAzF (plasmid #31186)[3], pEVOL_pBF (Plasmid #31190)[4] & pULTRA_CNF (plasmid #48215)[5,6] were provided by *Addgene* (USA), gifted by Prof. Peter Schultz. pBAD_GFP11_T7LysH17A (plasmid #59591) was also provided by *Addgene (*USA), deposited by Prof. Richard Kingston (Bulloch *et al*. 2014)[7]. pEVOL_NapA was recloned by Matúš Gajdoš at the Institute of Biotechnology, STUBA, based on the publication (Kolev *et al.*, 2014)[8]. pET41b_GFP (1-10) was ordered as the synthetic gene (*Genescript*, USA).

Supplementary Table 2. List of primers used in this study. Site of mutations are highlighted in red.

#	name	5'→3' seq	purpose
**1**	PFE_GFP_for	gcgactagtatgagcacatttgttgcaaaagacgg	Cloning into pBAD_GFP
**2**	PFE_GFP_rev	gaccgcttctgcgttctgatttaatctg	
**3**	225_fw	ggcgaccagatctagccgttcgagacc	Quikchange^{TM}-PCR
**4**	225_rv	ggtctcgaacggctagatctggtcgcc	
**5**	120_fw	ctggtgctgctgggctaggtcaccccgctg	
**6**	120_rv	cagcggggtgacctagcccagcagcaccag	
**7**	93_fw	accctggtgggctagtccatgggcggc	
**8**	93_rv	gccgcccatggactagcccaccagggt	
**9**	162_fw	ttcaacgcaccgtagtatggcatcaac	
**10**	162_rv	gttgatgccatactacggtgcgttgaa	
**11**	224_fw	gatggcgaccagtaggtgccgttcgag	
**12**	224_rv	ctcgaacggcacctactggtcgccatc	
**13**	158_fw	ttcatcagcgattagaacgcaccgttc	
**14**	158_rv	gaacggtgcgttctaatcgctgatga	
**15**	28_fw	ttcagccacggttagctactggatgcc	
**16**	28_rv	ggcatccagtagctaaccgtggctgaa	
**17**	198_fw	tgcgtcaccgcgtaggccgaaaccgac	
**18**	198_rv	gtcggtttcggcctacgcggtgacgca	
**19**	121_fw	ctgctgggcgcctagaccccgctgttc	
**20**	121_rv	gaacagcggggtctaggcgcccagcag	
**21**	253_fw	gcgccccacgggtaggcggtgacccac	
**22**	253_rv	gtgggtcaccgcctacccgtggggcgc	
**23**	PFE_seq_f	cgttcatctttccctggttgccaatg	sequencing primers
**24**	PFE_seq_r	gaccgcttctgcgttctgatttaatctg	

3. Supplementary Figures

Figure S1: SDS-PAGE analysis of the wtPFE and 2 variants (PFE_F198_NapA and PFE_V225_NapA) after large-scale expression and His-tag purification.

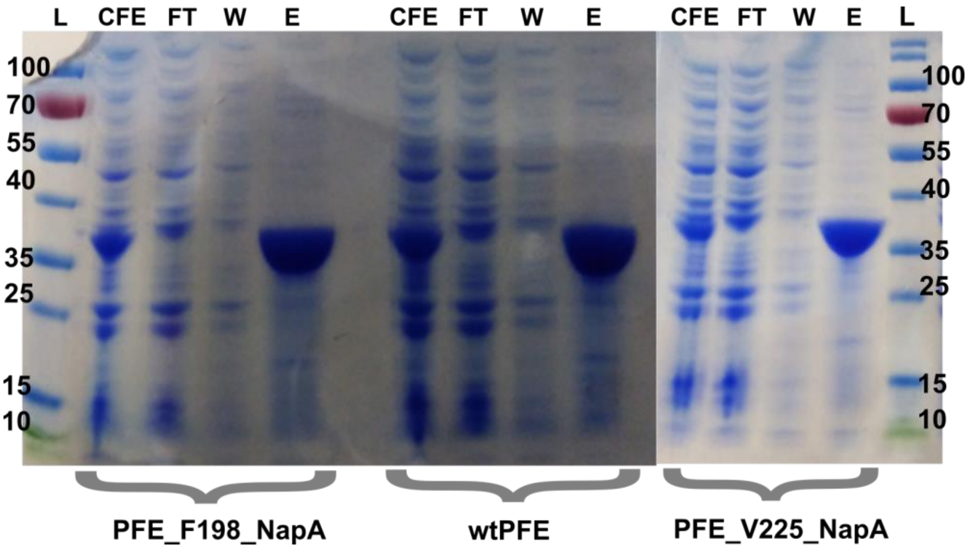

Legend: L - Ladder (PageRuler™ Prestained Protein Ladder, *Thermo Fisher Scientific*), CFE – Cell-free extracts, FT – Flow-through column, W - wash fraction, E - elution fraction

Figure S2: SDS-PAGE gels of the different PFE mutants after large-scale expression, purification and rebuffering.

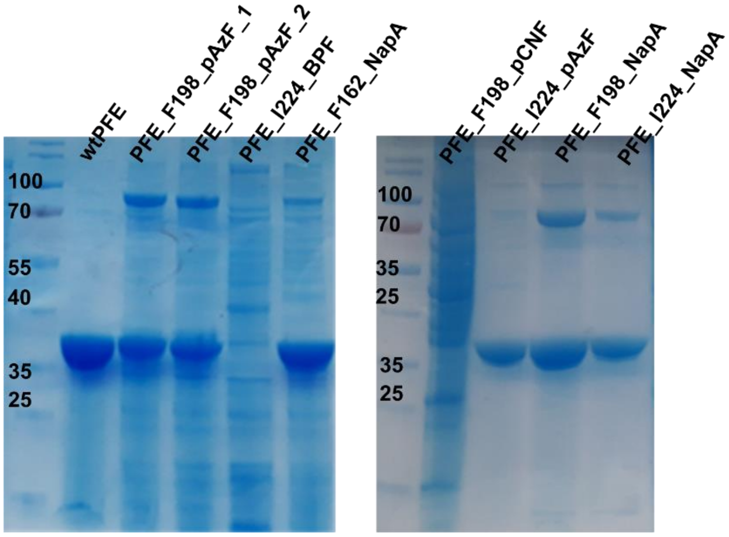

Results of MS-ESI

Figure S3. Mass spectra of wtPFE and chosen variants. All masses were observed with N-terminal methionine excision.

wtPFE Mass$_{Calc}$ (-Met) 34471.27 Mass$_{Obs}$.:34471.37

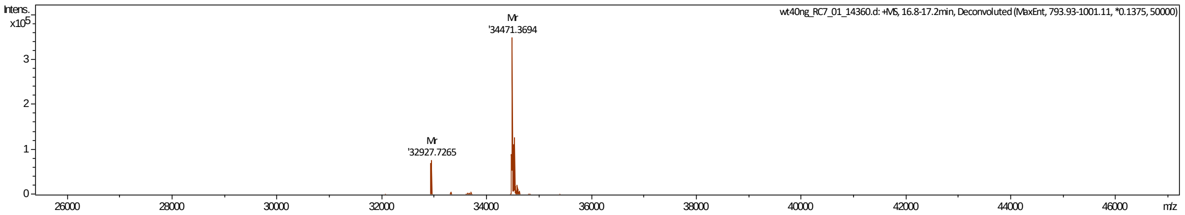

PFE_F162_NapA Mass$_{Calc}$ (-Met) 34521.30 Mass$_{Obs}$.:34521.40

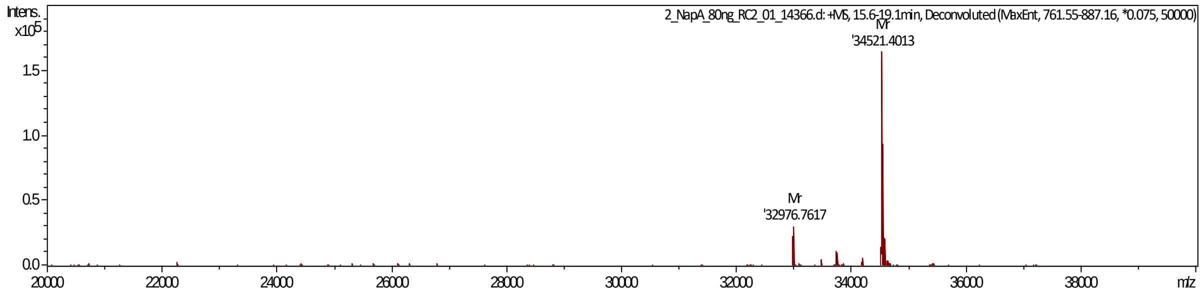

PFE_F198_pAzF Mass$_{Calc}$ (-Met) 34512.28 Mass$_{Obs}$.:34512.42

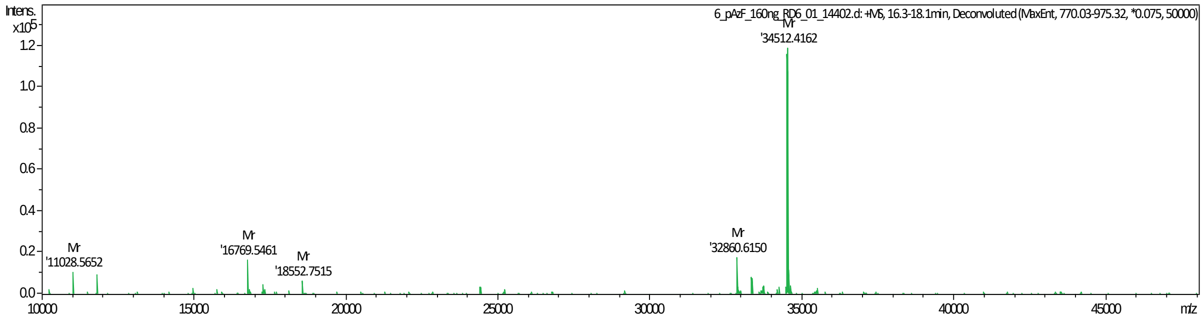

PFE_I224_NapA Mass$_{Calc}$ (-Met) 34555.28 Mass$_{Obs}$.:34555.28

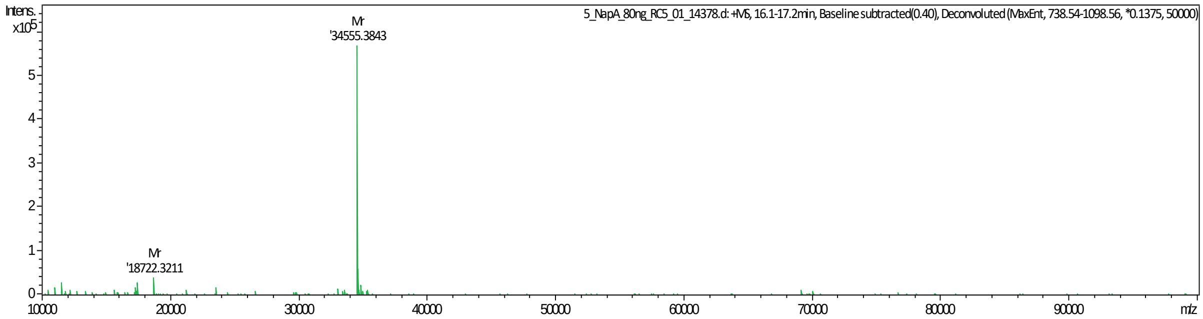

PFE_I224_pAzF Mass$_{Calc}$ (-Met) 34546.27Mass$_{Obs}$.:34546.36

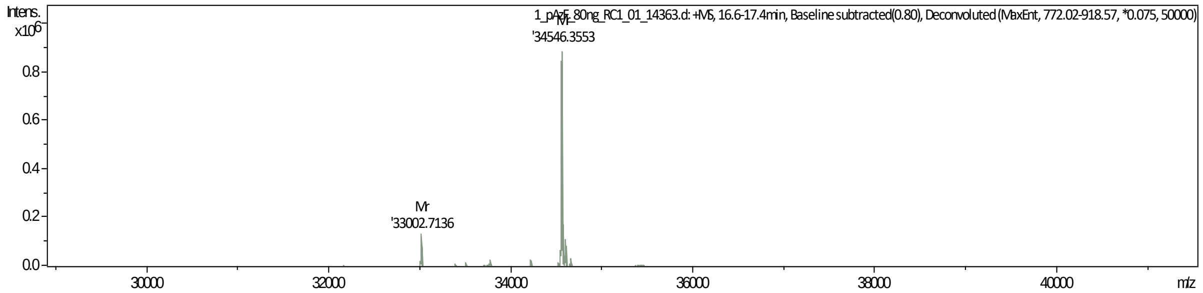

Split-GFP assay

Figure S4. Calibration curve of the split-GFP assay (measured at each split-GFP solution preparation)

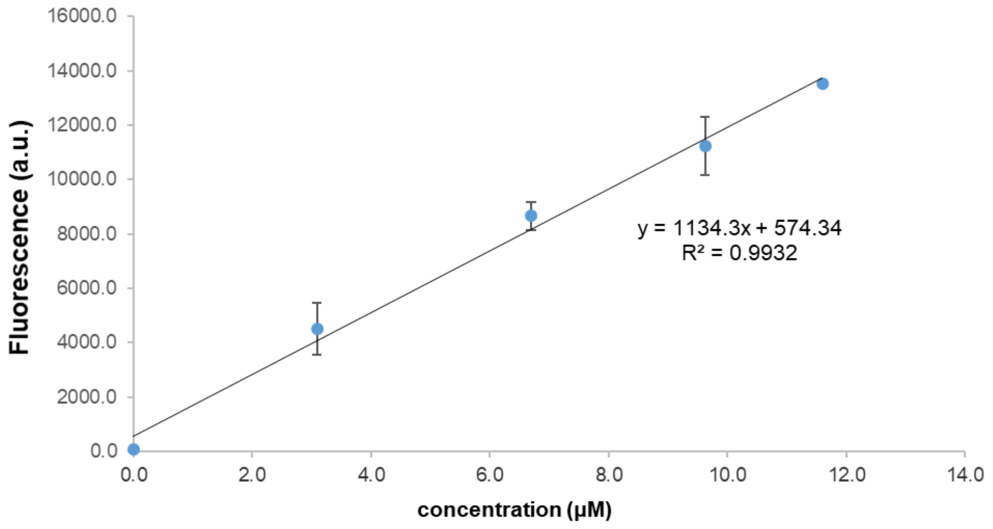

Figure S5. Bar graph comparison of expression levels of PFE variants measured in cell-free extracts (in µM) using the split-GFP assay and corresponding reaction rates measured for pNpA assay of the chosen PFE variants and wt.

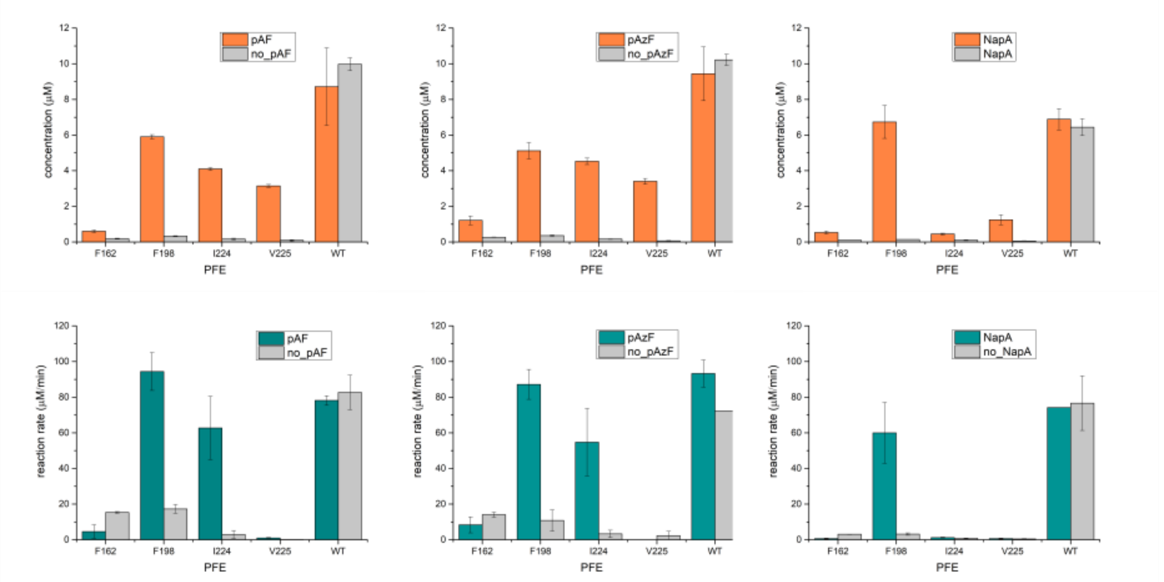

GC chromatograms

Figure S6. Chiral GC-FID analysis of results of the PFE-catalyzed of *rac*-ethyl 3-phenylbutyrate kinetic resolution.

PFE_F162NapA, 4h reaction time

<Chromatogram>

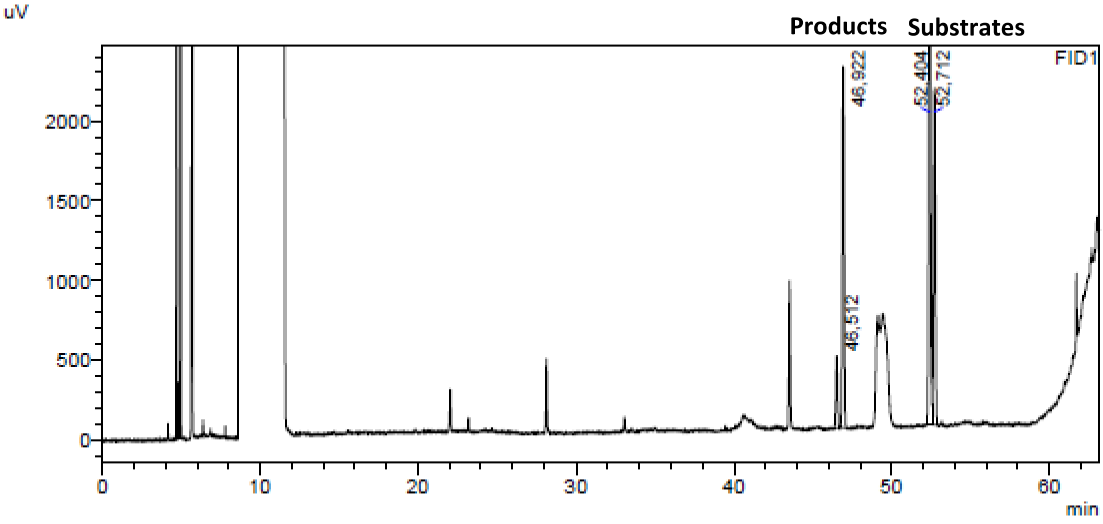

<Peak Table>

FID1

Peak#	Ret. Time	Area	Height	Conc.	Unit	Mark	Name
1	46,512	3307	448	0,000			
2	46,922	17129	2255	0,000			
3	52,404	26383	3184	0,000			
4	52,712	17313	2101	0,000		V	
Total		64132	7987				

PFE_I224pAzF, 4h reaction time

<Chromatogram>

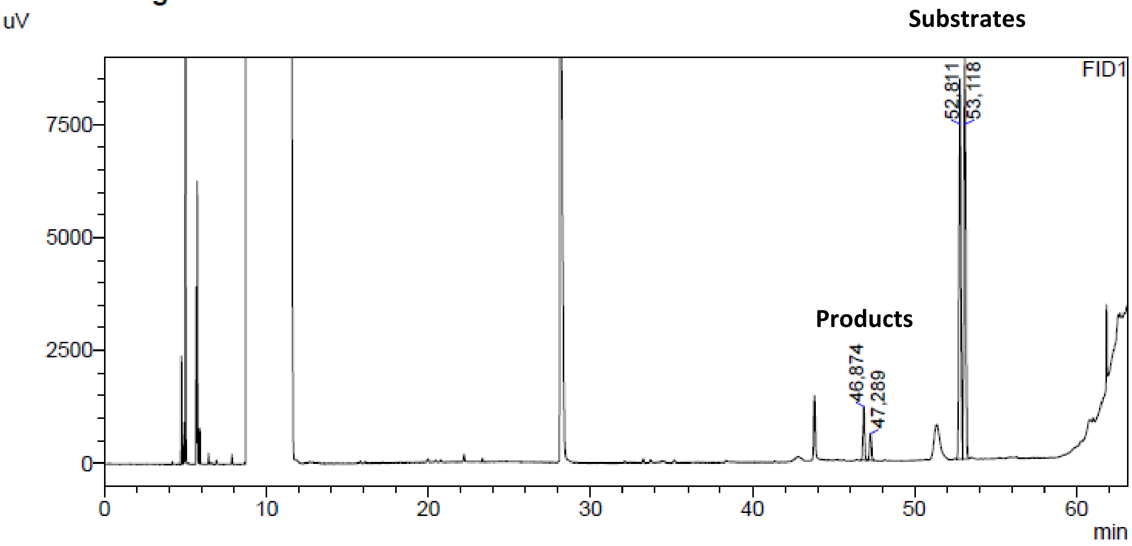

<Peak Table>

FID1

Peak#	Ret. Time	Area	Height	Conc.	Unit	Mark	Name
1	46,874	8851	1186	0,000			
2	47,289	4415	590	0,000			
3	52,811	68651	8410	0,000			
4	53,118	73412	8879	0,000		V	
Total		155329	19065				

References

[1] J. Santos-Aberturas, M. Dörr, U. T. Bornscheuer, in *Methods Mol. Biol.*, Humana Press Inc., **2018**, pp. 157–170.

[2] C. S. Chen, Y. Fujimoto, G. Girdaukas, C. J. Sih, *J. Am. Chem. Soc.* **1982**, *104*, 7294–7299.

[3] J. W. Chin, S. W. Santoro, A. B. Martin, D. S. King, L. Wang, P. G. Schultz, *J. Am. Chem. Soc.* **2002**, *124*, 9026–9027.

[4] J. W. Chin, A. B. Martin, D. S. King, L. Wang, P. G. Schultz, *Proc. Natl. Acad. Sci. U. S. A.* **2002**, *99*, 11020–11024.

[5] K. C. Schultz, L. Supekova, Y. Ryu, J. Xie, R. Perera, P. G. Schultz, *J. Am. Chem. Soc.* **2006**, *128*, 13984–13985.

[6] A. Chatterjee, S. B. Sun, J. L. Furman, H. Xiao, P. G. Schultz, *Biochemistry* **2013**, *52*, 1828–1837.

[7] E. M. M. Bulloch, R. L. Kingston, *Anal. Biochem.* **2014**, *465*, 53–62.

[8] J. N. Kolev, J. M. Zaengle, R. Ravikumar, R. Fasan, *ChemBioChem* **2014**, *15*, 1001–1010.
